# Transparent Rule Enablement Based on Commonization Approach in Heterogeneous IoT Edge Networks

**DOI:** 10.3390/s23198282

**Published:** 2023-10-06

**Authors:** Wenquan Jin, Yong-Geun Hong, Jaeseung Song, Jaeho Kim, Dohyeun Kim

**Affiliations:** 1 Department of Electronic & Communication Engineering, Engineering College, Yanbian University, Yanji 133002, China; jinwq@ybu.edu.cn; 2 Department of Artificial Intelligence & Convergence, Daejeon University, Daejeon 300716, Republic of Korea; yghong@dju.kr; 3 Department of Computer and Information Security, Sejong University, Seoul 05006, Republic of Korea; jssong@sejong.ac.kr; 4 Department of Electric Engineering, Sejong University, Seoul 05006, Republic of Korea; kimjh@sejong.ac.kr; 5 Department of Computer Engineering, Jeju National University, Jeju 63243, Republic of Korea

**Keywords:** edge computing, EdgeX, Internet of Things, Open Connectivity Foundation, proxy, rules engine, transparent computing

## Abstract

The paradigm of the Internet of Things (IoT) and edge computing brings a number of heterogeneous devices to the network edge for monitoring and controlling the environment. For reacting to events dynamically and automatically in the environment, rule-enabled IoT edge platforms operate the deployed service scenarios at the network edge, based on filtering events to perform control actions. However, due to the heterogeneity of the IoT edge networks, deploying a consistent rule context for operating a consistent rule scenario on multiple heterogeneous IoT edge platforms is difficult because of the difference in protocols and data formats. In this paper, we propose a transparent rule enablement, based on the commonization approach, for enabling a consistent rule scenario in heterogeneous IoT edge networks. The proposed IoT Edge Rule Agent Platform (IERAP) deploys device proxies to share consistent rules with IoT edge platforms without considering the difference in protocols and data formats. Therefore, each device proxy only considers the translation of the corresponding platform-specific and common formats. Also, the rules are deployed by the corresponding device proxy, which enables rules to be deployed to heterogeneous IoT edge platforms to perform the consistent rule scenario without considering the format and underlying protocols of the destination platform.

## 1. Introduction

The vision of industry 4.0 inspires various industrial domains to integrate heterogeneous technologies in a linear manner, for performing intelligence and autonomy in the smart spaces based on connected Internet of Things (IoT) edge devices. With the increasing number of IoT edge devices, the volume of data in these connected devices is growing rapidly. For reacting to events from the IoT edge environment based on data in real time or near real time, an event-driven mechanism can be included by the IoT applications to serve in the network edge, where the sensors and actuators operate to provide IoT edge services [1,2,3,4,5]. Rules are deployed in the network edge to operate service scenarios using the IoT edge nodes. The event-driven engine in the nodes filters events based on the deployed rule conditions to execute further actions, which supports the dynamic and autonomous operation of services in the edges of networks. Moreover, it enables more complicated processes, such as analyzation, recognition and prediction, and makes decisions automatically in the network edge. Complex event processing (CEP) solutions are performed in the IoT edge nodes with the dynamic rules to handle these processes, being based on data analysis and machine learning (ML) methods [6,7,8]. However, the heterogeneity of the platforms in the IoT edge networks brings challenges to operating the consistent rule scenario in the nodes to achieve the same purpose.

In the edge of the networks, enabling the interoperability of heterogeneous devices is difficult without configuring the same platform for each connected device. Nevertheless, satisfying the paradigm of the IoT in order to connect heterogeneous industrial devices is important. Many IoT edge standard frameworks try to provide interworking approaches for bridging the data based on proxy, gateway and middleware [9,10,11]. For delivering the data from one platform to another, the communication protocols and data formats should be considered, which are dependent on the frameworks. Most standards try to support unified interfaces to provide services. However, deploying the specialized platforms to industrial domains is necessary, due to the performance efficiency of satisfying the constrained environment including limited power supply and computing resources. Also, the variety of connectivity is important for connecting various devices through the specified networks, to develop various applications under emerging IoT-specific protocols as well as under traditional protocols [12,13,14]. To operate the consistent rule scenario in the rule-enabled IoT edge architecture, the heterogenous IoT edge devices must have an awareness of the rules of existing platforms and newly developed emerging solutions.

To provide autonomous and dynamic service scenarios in the heterogeneous IoT edge networks, we propose a transparent rule enablement based on the commonization approach to provide consistent rule operation using the IoT Edge Rule Agent Platform (IERAP). The proposed IERAP is deployed in the proxy layer to bridge clients and devices in heterogeneous IoT edge networks. Between different IoT edge networks, the heterogeneity of the platforms is handled by the IERAP in order to deliver the corresponding format of the rule profile to the destination platform through the platform-specific protocol. The IERAP includes the device proxies to enable the heterogeneous rule profiles to be in a common format and to be registered through the rule registry for the further deployment process. The device proxies of the IERAP translate rules between the platform-specific and the common format. To deploy rules to the heterogeneous IoT edge platforms, the device proxies translate the common format to a platform-specific style and deliver it to the corresponding platforms. In the IERAP, each device proxy only considers the corresponding IoT edge network in order to perform translation and deployment. Based on the IERAP, the heterogeneous IoT edge platforms operate the consistent rule scenario although heterogeneous rules are delivered to the environment.

The main contribution of this paper is summarized as follows.

In Section 2, related works are presented, including existing rule-based IoT edge frameworks and proxies for translating between heterogeneous protocols. Also, the standard IoT edge frameworks are introduced that have been adopted to implement the proposed rule-enabled IoT edge architecture.

In Section 3, the proposed IoT edge architecture is presented, which enables the rule-based service scenarios in heterogeneous IoT edge networks.

In Section 4, the details of the proposed IERAP are presented. Based on the IERAP, the rules are deployed and operated without considering the heterogeneity of the platforms in the IoT edge networks.

In Section 5, the development details and results of the proposed transparent rule-enabled IoT edge platform are presented.

In Section 6, the performance results of the proposed IERAP are presented including the initial memory usage of the deployed microservice modules, the network latency and memory usage in the deploying process.

Finally, we conclude our paper in Section 7.

## 2. Related Works

For bridging the platforms between heterogeneous IoT edge networks, the underlying protocols and transferring data formats are considered to enable the interoperability in the heterogeneous architecture. In the oneM2M-based platform, the interworking proxy application entity is deployed in the service layer to handle the messages to other framework-based platforms such as IoTivity and AllJoyn [15]. For bridging different network protocols in the framework, the OCF proposes the interworking proxy to include the implementation of bridged server and OCF client for handling the messages from a non-OCF client [16]. The EdgeX framework provides service-based microservices which enable multiple device proxies to be deployed in the EdgeX-based platform to handle corresponding platform-specific IoT edge networks [17]. Li et al. [18] proposed an OCF-based multi-protocol gateway to access non-OCF services based the centralized management service to support the semantic conversion scheme in the translation process. Esquiagola et al. [19] proposed an interception proxy including functions of transparent discovery communication between devices to extend the interaction capabilities of the IoT edge network for enabling the interoperability of heterogeneous communication protocols. According to these studies, the interworking proxy is the key to enable the interoperability of the heterogeneous network elements transparently based on protocol support and translation.

A rules engine is adopted by many IoT edge frameworks to enable the autonomous and dynamic operation of various service scenarios in the IoT edge platforms [20,21,22,23]. As a rule-based system, the CEP filters incoming events from sensors to trigger actions according to the defined rules. Based on the CEP architecture, oneM2M proposes the action-triggering mechanism (TR-0060-V-0.2.0) to trigger actions based on the configuration of conditions for autonomously sending a series of commands. The EdgeX framework uses a Kuiper rules engine to handle events in the edge of the networks using the JSON-based rule profile. In the OCF framework (OCF optional specification 2.2.3), the rules engine is performed by the OCF server that receives the sensing data to process based on a pre-deployed profile of rules and action. Luo et al. [24] proposed a scalable rules engine system in the IoT edge environment by embedding the Drools in the edge gateways for enabling automation in the IoT environment by the action-triggering mechanism. Choochotkaew et al. [25] proposed a fully distributed complex event-processing engine by deploying event-based and stream-based approaches to the IoT edge devices to handle the real-time data in the edge of the networks. Chen et al. [26] proposed a Spark-based rules engine that filters the IoT real-time streaming data based on Drools-style rules. For supporting the rule interoperability in heterogeneous rule-enabled IoT edge platforms, a protocol and data translator are required that bring a variety of IoT edge frameworks to the development environment.

Transparent computing is a paradigm that enables clients to request services without considering underlying data pipelines, protocols and data formats from the heterogeneous IoT edge networks [27,28,29]. Many IoT edge application domains leverage the paradigm to enable the transparency between devices to deliver data for providing seamless services. For management of IoT devices, Hui et al. [30] proposed a centralized IoT architecture to provide transparent management to support cross-platform and scalable IoT applications. The centralized architecture is able to integrate multiple solutions to support various interfaces based on the sufficient computing resources of the platforms such as cloud computing [31,32,33]. However, due to the communication latency and data privacy, the computing resources are deployed close to the environment where the device platforms are configured for a constrained environment.

The interworking proxy can be deployed in the network edge or integrated into the device for supporting the communication between different protocols [34,35,36]. In our previous work [37], the proxy was used for bridging different protocol-based platforms in the network edge. Through the device registration, the service resources of the proxy are mapped with the destination IoT edge services which enables clients to access the IoT edge services transparently. In the proposed rule-enabled IoT edge architecture, the rule profile as data is delivered between heterogeneous IoT edge platforms based on the interworking proxy. In our previous work [38], we proposed a transparent rule deployment approach based on the rule translator which was embedded in a specific platform to enable the rule translation in different platforms. Therefore, compared with our previous work, the proposed approach in this paper deploys device proxies to a specific platform for enabling multiple rule translators dynamically in the heterogeneous platforms without modifying the platforms.

## 3. Transparent Rule-Enabled IoT Edge Architecture

**Figure 1:**
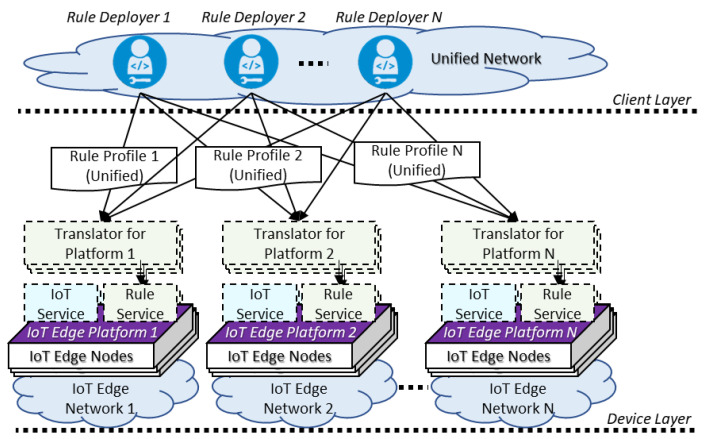
Ideal standard rule enablement architecture in heterogeneous IoT edge networks.

For enabling the consistent rule-based service scenario in the heterogeneous IoT edge networks, the same knowledge of the rule context must be deployed in each IoT edge platform. As shown in Figure 1, the translators can be included in the platform for converting the incoming rule context to the platform-specific format. The translator also provides the communication protocol to the rule deployers to receive the rule context. Therefore, the translators in the platforms provide a consistent interface to the rule deployers. This process can be a standard rule enablement architecture in heterogeneous IoT edge networks. As an ideal standard, the architecture requires a unified rule profile format and communication protocol for receiving the rule context from the rule deployer. Then, the rule context is converted and delivered to each platform of the specific IoT edge network. Based on the ideal standard, the existing frameworks can equip the translator to receive the unified rule profile through the unified communication protocol and convert the rule profile from unified to platform-specific for deploying to the specific IoT edge platforms.

The ideal standard rule enablement architecture requires translators in all platforms for translating the unified rule profile which also requires the communication modules for receiving the rule profile through the unified communication protocol. However, for providing services in the constrained environment, the IoT edge platforms struggle to equip the general communication modules such as Wi-Fi or Ethernet to support HTTP over the IP. Also, the existing clients are required to be updated to send the unified rule profile through the unified communication protocol for synchronizing the rule context in the heterogeneous IoT edge networks. Therefore, the rule deployment clients need to consider whether the destination platform is developed using the same IoT edge framework or not.

**Figure 2:**
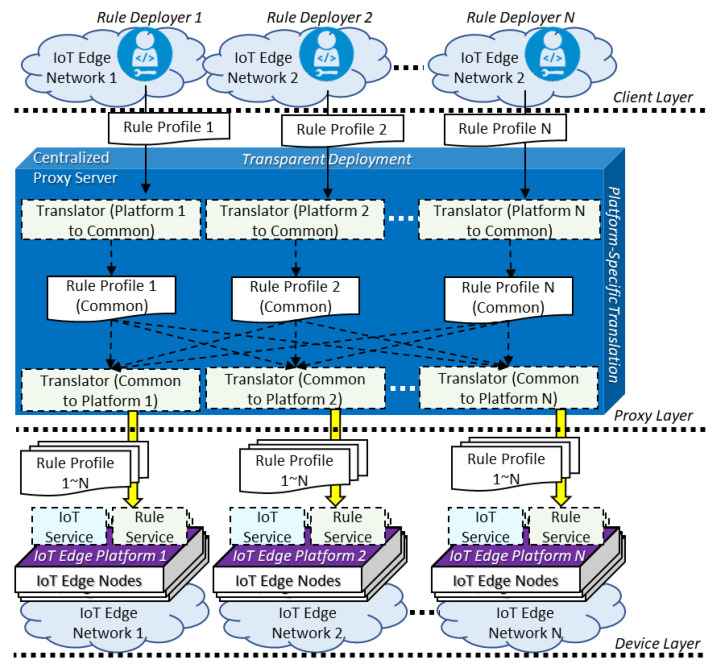
Proposed transparent rule enablement architecture based on commonization approach.

For operating the consistent rule-based service scenario without updating the existing IoT edge platforms, as shown in Figure 2, we propose the transparent rule enablement architecture that includes the proxy layer between client and device layers to bridge the rule deployers and platforms in heterogeneous IoT edge networks. The rule deployers are included in the client layer to access the IoT edge nodes that are included in the device layer. For using the same platform in both client and device, the rule deployer can directly deploy the consistent rule context to the nodes. However, interaction between different platforms requires the bridge to enable interoperability in the heterogeneous IoT edge networks.

In the proxy layer, the centralized proxy server provides translating approaches between client and device layers to deliver the consistent rule knowledge to the heterogeneous networks. We propose the commonization approach to perform the translating mechanism between platforms. In the proxy server, the translator receives the rule profile from the rule deployer and translates the rule context from the platform-specific format to the common format. Then, the common format of the rule context is translated to the platform-specific format for the destination IoT edge network. Using the commonization approach, the platform-specific rule deployers can deploy the consistent rule knowledge to the heterogeneous IoT edge nodes without considering the underlying protocol and rule framework of the destination platform. Also, each translator is deployed to serve the platform-specific protocol.

**Figure 3:**
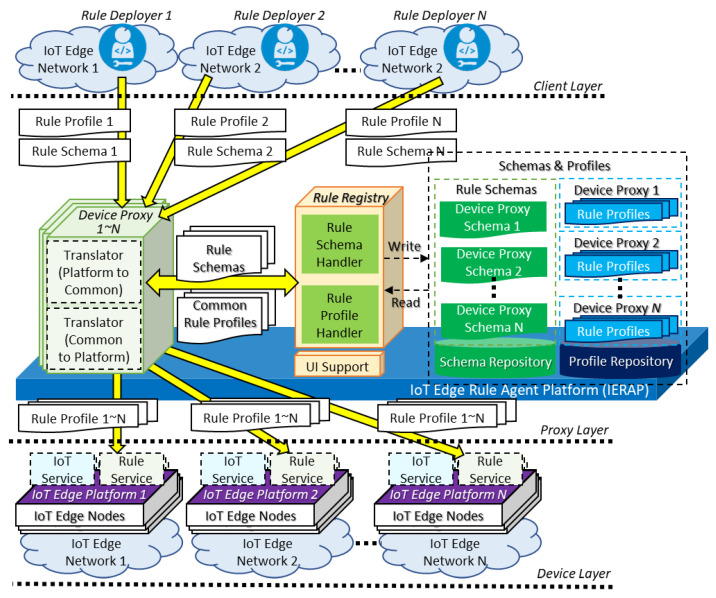
Proposed IERAP for transparent rule enablement architecture.

For enabling the transparent deployment and platform-specific translation to operate the consistent rule in heterogeneous IoT edge networks, the IERAP is proposed to provide corresponding interfaces to the client layer and delivers the rule knowledge to the device layer. Figure 3 presents the proposed IERAP architecture that is included in the proxy layer to bridge the client and device layer. In the proxy layer, the IERAP delivers the corresponding format of rules to the destination IoT edge platforms through the platform-specific protocol. The IERAP includes four parts which are device proxy group, rule registry, rule repositories and UI support. A device proxy is the entry of the IERAP that receives the schema and rule profiles through the platform-specific protocol and translates the platform-specific format of rule context to the common format. Also, the device proxy delivers the rule profile to the nodes through the platform-specific protocol based on translating the common format to platform-specific format.

Multiple device proxies are included to handle multiple IoT edge networks. In the translating process, the device proxy includes the translators of both sides to translate platform-specific to common and common to platform-specific. Also, the device proxy includes the server to receive the rule profile and rule schema through the platform-specific protocol. The rule schema is used for configuring the rule deployment to an IoT edge network. The derived common rule profiles and rule schemas are stored in the repository and retrieved through the rule registration. The UI support is used for providing the client services to present the details of rule deployment based on the rule schemas and profiles.

## 4. IoT Edge Rule Agent Platform in IoT Edge Architecture

The proposed transparent rule-enabled IoT edge architecture is performed based on the IERAP that is serviced between rule deployers and IoT edge nodes to provide schema and rule registration, transparent rule deployment and operation and client services.

### 4.1. Rule Schema Registration

**Figure 4:**
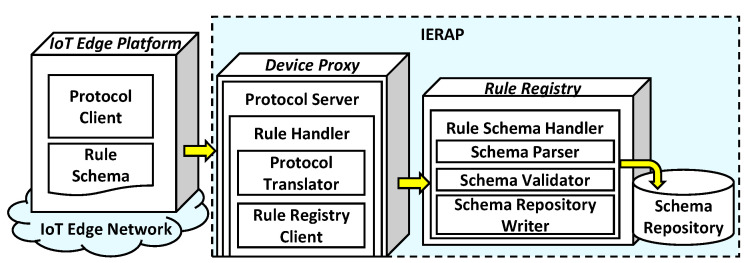
Rule schema registration model.

The rule schema is a medium for illustrating the rule profile and corresponding IoT edge network. As shown in Figure 4, the rule schema is deployed by an IoT edge platform that sends the schema from an IoT edge network to the IERAP using the protocol client. The IoT edge platform can be a device that can be used for operating the rule scenario by deploying the rule to other devices in the same network as well as different networks.

Whether the network is the same or different, the IERAP receives the rule schema through the device proxy and delivers it to the rule registry using the rule registry client. For each network, a device proxy is deployed to bridge the IoT edge platforms and IERAP. The device proxy includes the protocol server to handle the messages from the corresponding network. The protocol translator is necessary to convert a protocol for the IERAP. Through the translator, the IERAP receives messages and delivers them to the internal services. The rule registry client is used for delivering the rule schema to the rule schema handler of the rule registry. For storing the rule schema in the schema repository, the rule schema handler includes a parser, validator and repository writer to handle the schemas.

**Figure 5:**
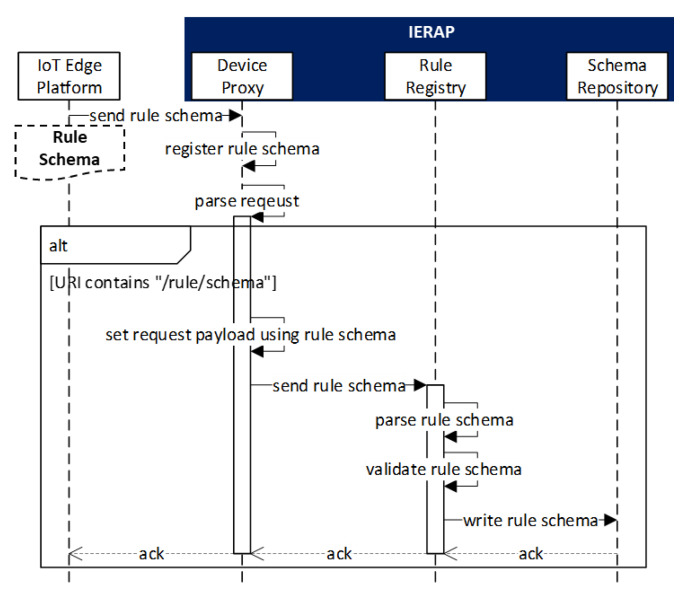
Rule schema registration scenario.

Figure 5 shows the sequence of the rule schema registration scenario through the interactions between IoT edge platform and IERAP.

The IoT edge platform sends the rule schema to the device proxy that performs the registration process using the registration client. The rule registry provides the internal services that are used by the registration client in the IERAP. The URI/rule/schema is identified to access the service for registering the rule schema. In the device proxy, the message is translated from the protocol of the IoT edge platform, then the client forwards the translated rule schema message to the rule registry. Once the registry receives the rule schema, the schema parser and validator are used to extract and validate the information from the schema and the schema is saved to the repository using the schema repository writer.

**Figure 6:**
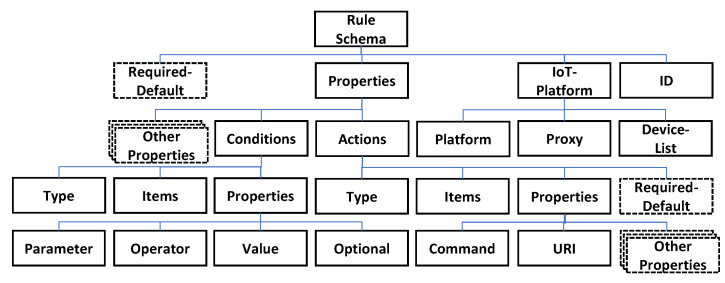
Rule schema data structure.

The rule schema is developed based on a JSON schema that is used for explaining the JSON profile. As shown in Figure 6, the rule schema data has five layers. The first layer includes the root that presents the type of data. In the second layer, the required-default attribute is used for presenting the properties of the rule profile that must be declared in the first layer. For heterogenous IoT edge networks, the required default is different.

The properties attribute includes conditions and actions by default. According to the required default, the properties attribute includes more properties for the corresponding IoT edge network. The IoT-platform attribute includes the platform, proxy and device list where the platform presents the name of the corresponding IoT edge platform, the proxy presents the URI of the device proxy of the IERAP and the device list presents the URI of the devices that are deployed in the IoT edge network. The conditions and actions attributes include type, items and properties that are used for interpreting the JSON data. The conditions attribute includes parameter, operator, value and optional that are used for presenting a list of rule conditions. The actions attribute includes command and URI that are used for presenting a list of rule actions. The actions attribute also includes the required default that is used for extending the properties.

### 4.2. Transparent Rule Profile Deployment and Operation

In the rule-enabled heterogeneous IoT edge networks, a rule context is difficult to deploy using a consistent interface to different platforms that are developed based on different IoT edge frameworks. Therefore, operating the consistent rule scenario in multiple platforms is difficult because the rule knowledge may be reduced according to the platform-specific requirement which constrains the properties in the rule profile. The proposed IERAP is deployed in the proxy layer to bridge the client layer and the device layer for deploying the consistent rule knowledge in heterogeneous IoT edge networks.

**Figure 7:**
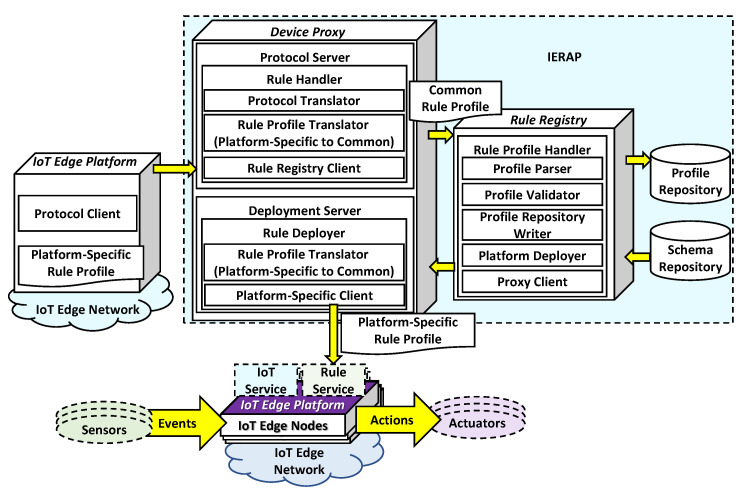
Rule profile deployment and operation model.

Figure 7 presents the rule deployment and operation model based on the data flow and functional architecture. For delivering the rule profile to the IoT edge platform and operate, the rule deployer includes the protocol client to send the platform-specific rule profile to the proposed IERAP. The rule deployer is an IoT edge platform that is deployed in a specific IoT edge network and sends data through the specific protocol. For receiving the data from the specific protocol, the IERAP includes the device proxy to handle the data of the specific protocol using the protocol server. In the protocol server, the rule handler provides protocol translation and rule profile translation using the translator functions. The rule profile translator is used for translating the rule profile from platform-specific to common. Then, through the rule registry, the translated common rule profile is stored in the repository. A rule profile is delivered to the IERAP and the rule registry activates the rule deployment process based on the deployment server.

The deployment server includes the rule profile translator that is used for translating the rule profile from common to platform specific. The rule profile translator enables the device proxy to handle rule deployment for the specific platform through the interoperability between platform-specific and common rule profile. In this process, the rule schema is referred to by the rule registry to deploy the rule profile through multiple device proxies. The platform-specific client is used for delivering the rule profile that is translated from the common format for the specific platform.

**Figure 8:**
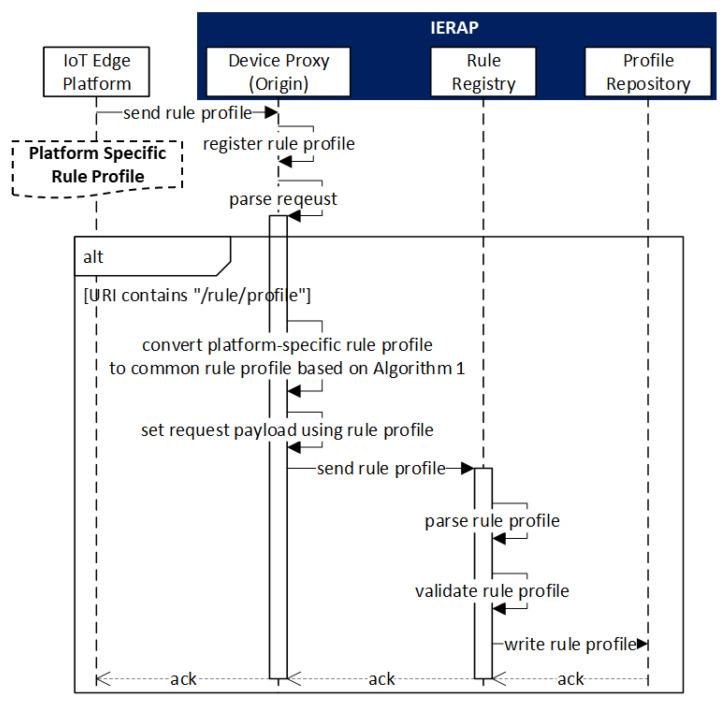
Rule profile registration scenario.

Figure 8 presents the registration scenario of the rule profile through the interaction between the IoT edge platform and IERAP.

The IoT edge platform has the platform-specific rule profile that is used for sending to the IERAP where the device proxy receives the profile and registers to the rule registry. The device proxy is developed for the IoT edge platform, therefore, the received profile is converted to the common format using the proposed Algorithm 1. Then, the profile is delivered to the rule registry and saved to the profile repository. In this process, the device proxy is associated with the IoT edge platform that is a client to perform the rule profile deployment. Therefore, the device proxy is enabled to receive and parse the rule profile through the same protocol and IoT edge framework.
**Algorithm 1: translateSpecific2Common**1 2 3 4 5 6 7 8 9 10 11 12 13 14 15 16 17 18 19 20 21 22 23 24 25 26 27**INPUT**: platform-specific **OUTPUT**: profile platform ← current platform ID; profile ← initialize to JSON object node; conditions ← initialize to JSON array node; actions ← initialize to JSON array node; ruleConditionList ← parse platform-specific condition expression to RuleConditionList; **while** *ruleConditionList.hasNext()* **do**  condition ← initialize to JSON object node;  ruleCondition ← ruleConditionList.next();  condition.put(“parameter”, ruleCondition.getParameter());  condition.put(“operator”, ruleCondition.getOperator());   condition.put(“value”, ruleCondition.getValue());  condition.put(“option”, ruleCondition.getOption());   conditions.add(condition); **end** ruleActionList ← parse platform-specific actions to RuleActionList; **while**
*ruleActionList.hasNext()*
**do** action ← initialize to JSON object node; ruleAction ← ruleActionList.next(); action.put(“uri”, ruleAction.getUri()); action.put(“command”, ruleAction.getCommand()); actions.add(action); **end** profile.set(“conditions”, conditions); profile.set(“actions”, actions); profile.set(“platform”, platform); profile.set platform-specific properties with values; return profile;

Algorithm 1 is used in the rule registration scenario for translating the rule profile from platform-specific to the common style. The input of the algorithm is the rule profile for the platform-specific style that is sent from an IoT edge platform. The output is the common style rule profile that is delivered to the rule registry.

For the common rule profile, the required properties are platform, profile, conditions and actions that are assigned by obtaining the value from the JSON data of the rule profile. The conditions and actions obtain the values from the JSON arrays that are converted to the list of the rule condition entity and rule action entity. Using the loop operation, the arrays are iterated to create the entities. In the iteration processes, the property conditions and actions are created and assigned to the common rule profile in the further process. At the end of the algorithm, the common profile includes the platform-specific properties that are used for the platform as well as other platforms if the properties are required.

**Figure 9:**
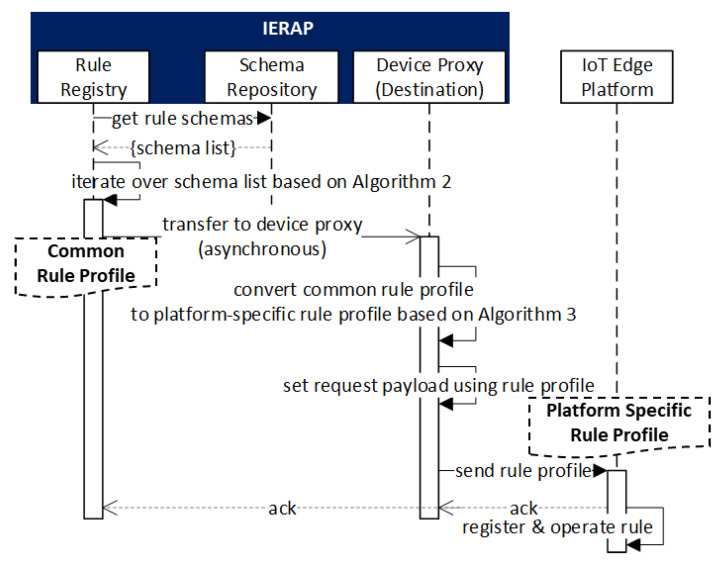
Rule profile deployment scenario.

Figure 9 presents the scenario of rule profile deployment to the IoT edge platforms using the IERAP. Once the rule registry receives the rule profile from the rule deployer through the device proxy, the rule registry saves the profile to the repository and deploys the profile to the IoT edge platform based on the rule schema. Each schema is used for providing the reference of the corresponding IoT edge network. The rule registry retrieves the rule schema list from the repository and iterates over the schema list based on Algorithm 2. Inside of the iteration, also, the device list of a schema is iterated. In the iteration of the device list, the common rule profile is transferred to the device proxy that converts the common rule profile to the platform-specific rule profile based on Algorithm 3. Then, the converted rule profile is delivered to the IoT edge platform that operates the rule based on the platform-specific rule profile.
**Algorithm 2: iterateOverSchemaList**1 2 3 4 5 6 7 8 9 10 11 12 13 14 15 16 17**INPUT**: profile, platform schemaIdList ← getSchemaIdList(); while *schemaIdList.hasNext()*
**do**  schemaId ← schemaIdList.next();  schema ← getSchemaJsonById();  proxy ← schema.get(“iot-platform”).get(“proxy”);  platform ← schema.get(“iot-platform”).get(“platform”);   deviceList ← schema.get(“iot-platform”).get(“device-list”);  **while**
*deviceList.hasNext()*
**do**    device ← deviceList.next();    profile.set(“uri”, device);     doAsynchronousRequest() {         uri ← ”http://localhost:” + proxy + ”/dp/” + platform;        payload ← profile;        request to uri with payload;    }  **end** **end**

Algorithm 2 is used for iterating over the schema list to obtain the information of destination platforms. The profile is the input of the algorithm that is a common rule profile and used in the payload of the request to the device proxy. The algorithm is performed by the rule registry once the schema list is retrieved from the schema repository.

In the process of the iteration over the schema list, by parsing each schema item, the values are assigned to the variables proxy, platform and deviceList that are used in the further iteration. Then, the iteration is operated over the deviceList to generate the request to the IoT edge platforms of each IoT edge network. In this process, for delivering the profile to each destination device, the URI of the device is included to the profile, and the profile is included in the payload of the request. The URI of the request comprises the device proxy information including the port and URI path for delivering the common profile to the corresponding device proxy to translate it to the platform-specific style.

Algorithm 3 is used in the process of converting the rule profile from the common style to the platform-specific style. The process is performed by the device proxy for each deployment of the rule profile based on parsing and converting. The device proxy is used for the IoT edge network which requires the rule profile to be suitable for the rule model of the platforms. For each IoT edge network, the IERAP include the corresponding device proxy to bridge the rules to the corresponding IoT edge platforms. The input of the algorithm is the common rule profile as the parameter name profile. The output is a platform-specific data entity that is further processed to be the payload of the deployment request.
**Algorithm 3: translateCommon2Specific**1 2 3 4 5 6 7 8 9 10 11 12 13 14 15 16 17 18 19 20 21 22 23 24 25**INPUT**: profile **OUTPUT**: platform-specific data entity ruleConditionList ← parse profile condition expression to RuleConditionList; **while** *ruleConditionList.hasNext()* **do**  ruleCondition ← ruleConditionList.next();  handle platform-specific conditions;  **if** *required properties are null* **then**    required properties ← default values;  **else**    required properties ← profile values;  **end** **end** ruleActionList ← parse profile actions to RuleActionList; **while** *ruleActionList.hasNext()* **do**  handle platform-specific actions;  **if** *required properties are null* **then**    required properties ← default values;  **else**     required properties ← profile values;  **end** **end** **if** *required properties are null* **then**   required properties ← default values; **else**  required properties ← profile values; **end** return platform-specific data entity;

Once the common rule profile is delivered to the corresponding device proxy, the device proxy translates the rule profile to the platform-specific style for the IoT edge platform. Firstly, the rule conditions are handled to assign the conditions values according to the platform-specific style by iterating over the conditions list of the common rule profile. Then, the rule actions are handled to assign the actions values according the platform-specific style by iterating over the actions list of the common rule profile. In the process, the required properties are also assigned in the sub-level of the actions by default values or profile values. The default values are presented in the rule schema that can be referred to when assigning the required properties. Also, the values can be assigned by the program code in the implementation.

**Figure 10:**
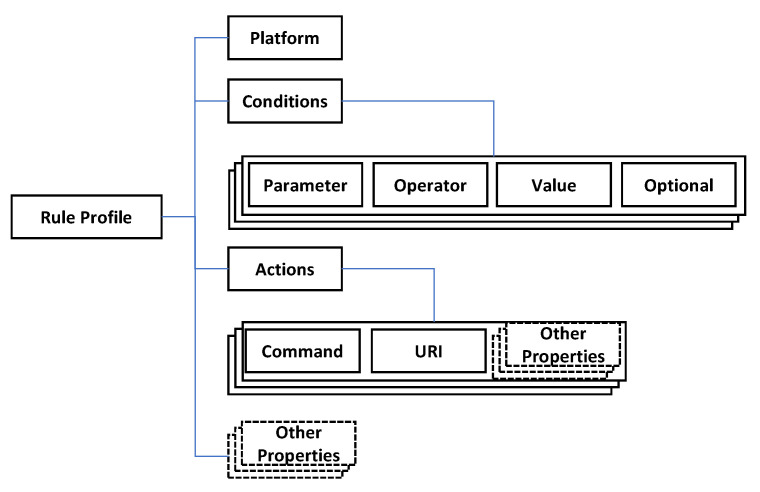
Rule profile data structure.

According to the rule schema, the rule profile is structured as shown in Figure 10. The rule profile is JSON data that include the properties platform, conditions and actions and other required properties. The platform property has a value that indicates which IoT edge network the rule profile is used for. In the conditions property, the properties parameter, operator and value are used for making an expression like “temperature” > 22. The optional property presents the logical operation to enable the set of the expressions using “or” and “and” operations. In the actions property, the properties command and URI are used for performing the actions through sending a command to an actuator. Also, other properties can be included in the rule profile for specific platforms.

### 4.3. Client Services for Accessing Rule Schemas and Profiles

**Figure 11:**
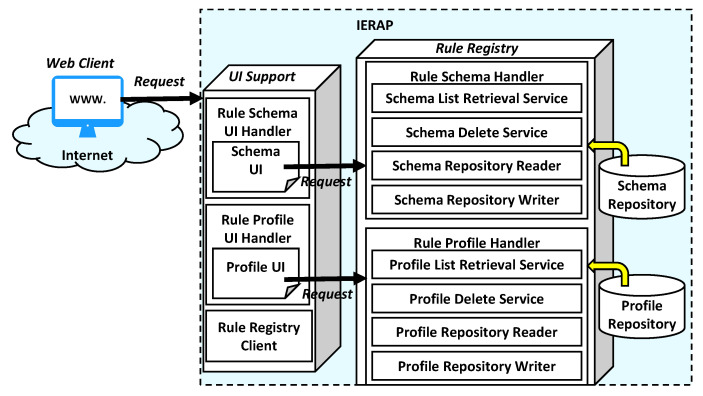
Client service model.

For presenting the information of the rule schemas and profiles to clients, the rule registry delivers the data through the APIs that are provided by the internal services of the rule schema handler and rule profile handler. The handlers access the repositories to write and read rule schemas and profiles. As shown in Figure 11, the services are used for retrieving and deleting the schemas and profiles using the repository reader and writer.

The UI support is a service provider that provides the UIs to the web clients. The web client application can work on web browsers to present the rule schemas and profiles. The UIs integrate the information of rule schemas and profiles with the delete function to handle each item of the retrieved list. For integrating the information with the UIs, the UI support sends requests to the handlers to obtain the information from the repository. Also, the delete function is presented with the identifier of a schema or profile to delete the selected schema or profile through the rule registry.

The UI support is not a necessary module for enabling transparent rule operation in heterogenous IoT edge networks. However, the exposed services and UIs support the administrators to manage the deployment status of the IERAP.

## 5. Development Details and Results

As presented in Table 1, for experimenting with the proposed transparent rule enabled IoT edge networks, we developed an EdgeX-based network service, OCF-based network service, rule registry and UI support to provide services in the IERAP. Also, an EdgeX-based rule engine and OCF-based rule server are deployed to provide rule services for performing the rule operation mechanism in IoT edge networks based on EdgeX and OCF. For testing the REST APIs, we use the Talend API test as the web client to request the microservice modules.

The modules of the IERAP interact through the microservices that are implemented based on Jettry in the rule registry and device proxies. The UI support is developed using Spring Boot to provide UIs to present the registered rule schemas and profile. The HTTP client is used to access the microservice in the IERAP, Jackson is used for implementing the JSON data reader and writer. The EdgeX-based rules engine is a part of the EdgeX-based IoT edge platform that is deployed in the EdgeX-based IoT edge network. The EdgeX platform is configured based on the EdgeX framework with the Hanoi version and EMQ Kuiper is used for operating the rules in the platform. For developing the OCF-based platforms, IoTivity 2.2.2 is included to implement the OCF services in the IoT edge platforms in the OCF-based IoT edge network. The rule server is included in the platform to provide the operation of the rules. The IERAP and EdgeX-based IoT edge platform are operated on the Raspberry Pi device based on the Ubuntu server. The OCF-based IoT edge platform is deployed in the Windows machine that emulates multiple nodes for collecting the performance.

For experimenting with the proposed IoT edge architecture and observing the performance of the IERAP, we configured two types of rule deployment scenarios. The first scenario is deploying a single device proxy for an OCF device network where multiple OCF-based rule servers are deployed. The second scenario is deploying multiple device proxies for multiple OCF device networks and each network includes an OCF-based rule server.

**Figure 12:**
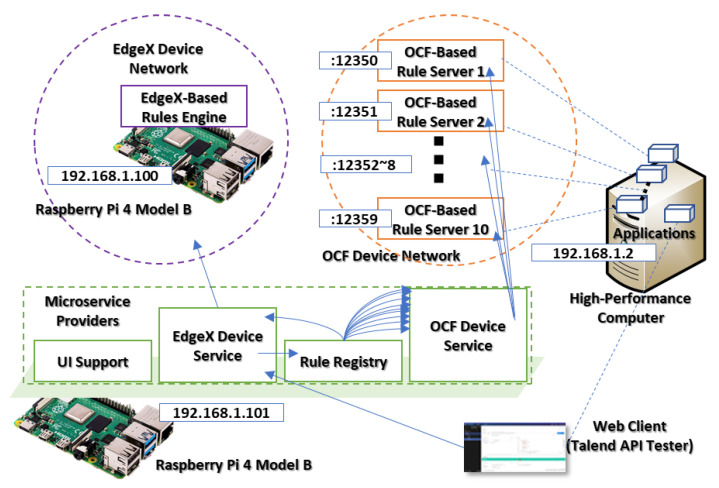
Single device proxy experiment.

Figure 12 presents the network environment of the first scenario that deploys an OCF device service module in the Raspberry Pi 4 Model B device to perform the device proxy of the IERAP. As a device proxy, the OCF device service translates the rule profile from common style to the OCF-based platform-specific style and deploys to the OCF-based rule servers. The PC emulates 10 OCF-based rule servers for an OCF IoT edge network. Therefore, the OCF device service delivers the platform-specific style rule profile to these OCF platforms. The scenario is started by the web client that deploys the EdgeX-based rule profile to the device where the EdgeX device service receives the profile and translates it to the common format. Then, the device proxy delivers it to the rule registry. Based on the registered rule schema, the rule registry delivers the common rule profile to the OCF device service for deploying to the destination IoT edge platforms. 

**Figure 13:**
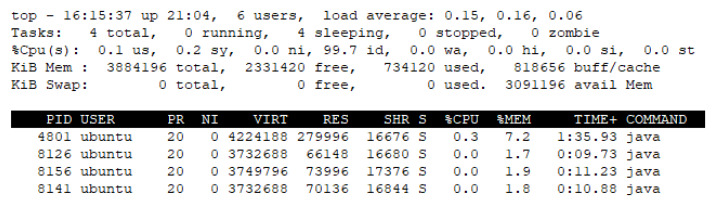
IERAP running status for single device proxy.

The rule schema for the OCF device network includes the URIs of these 10 devices that require the rule profile requests to the OCF device service 10 times for deploying the rule profile. Also, the rule profile delivers the common rule profile to the EdgeX device server for deploying the EdgeX-based rules engine. As shown in Figure 13, the IERAP include four modules that are identified by the PIDs. PID 4801 is the UI support, PID 8126 is the rule registry, PID 8141 is the EdgeX device service and PID 8156 is the OCF device service.

**Figure 14:**
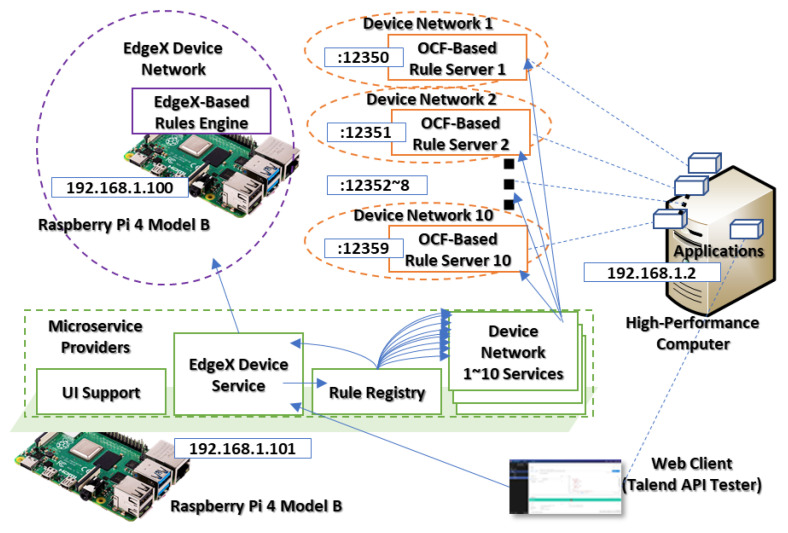
Multiple device proxy experiment.

Figure 14 presents the network environment of the second scenario that deploys multiple OCF device service modules in the device to perform multiple device proxies of the IERAP. The PC emulates 10 device networks and each network includes an OCF platform. Each device network service handles the corresponding OCF platform for deploying the platform-specific rule profile.

**Figure 15:**
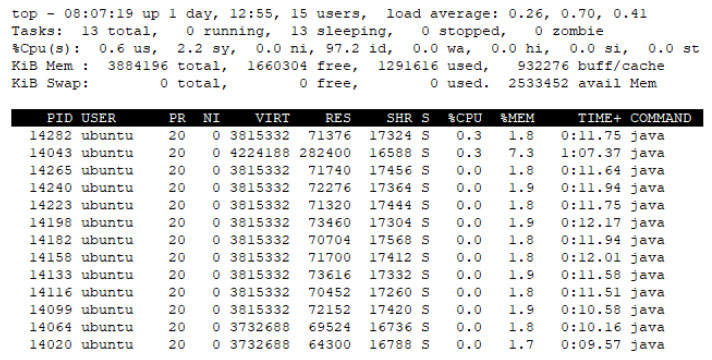
IERAP running status for multiple device proxies.

As shown in Figure 15, the result of the IERAP running status depicts that multiple device proxies are included in the IERAP for handling multiple device networks. PID 14020 is the rule registry, PID 14043 is the UI support and PID 14064 is the EdgeX device service that is used for receiving the EdgeX-specific rule profile and translating it to the common format. Then, the rule registry delivers the 10 device proxies to translate and deploy them to the 10 device networks.

**Figure 16:**
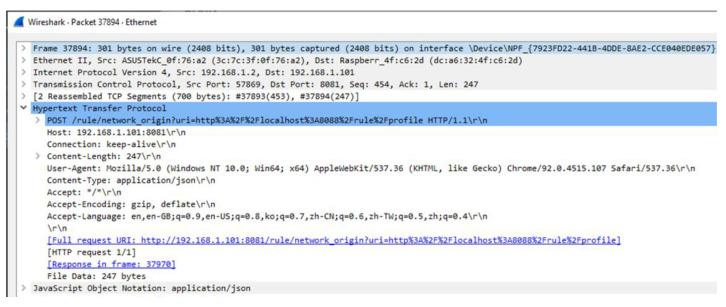
Network packet of rule profile deployment by EdgeX-based rules engine to IERAP.

Figure 16 shows the implementation result of the rule profile deployment by the EdgeX-based rules engine to the IERAP. The result is collected using Wireshark that is a tool to capture network packets. The tool is configured in the Windows computer where the EdgeX-based rules engine is deployed and sends the EdgeX-based rule profile to the IERAP. In the IERAP, the EdgeX device service receives the rule profile and translates it to the common rule profile. The captured network packet includes the EdgeX-based rule profile that is 247-byte JSON data.

**Figure 17:**
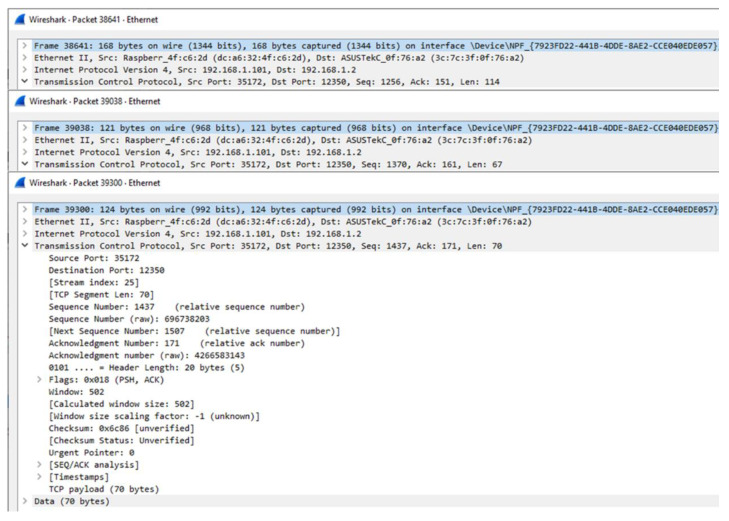
Network packet of rule profile deployment by IERAP to OCF-based rule server.

Figure 17 shows the implementation result of the rule profile deployment by the IERAP to the OCF-based rule sever that is deployed in the Windows computer to be captured by Wireshark. The device proxy for the OCF-based IoT edge network is the OCF device service and the device network services, respectively, in the two experiments. The device proxy in the IERAP translates the common rule profile to the OCF-based rule profile that comprises three parts and sends them to the OCF platforms separately. The process is performed for OCF platforms and supported by the device proxy.

**Figure 18:**
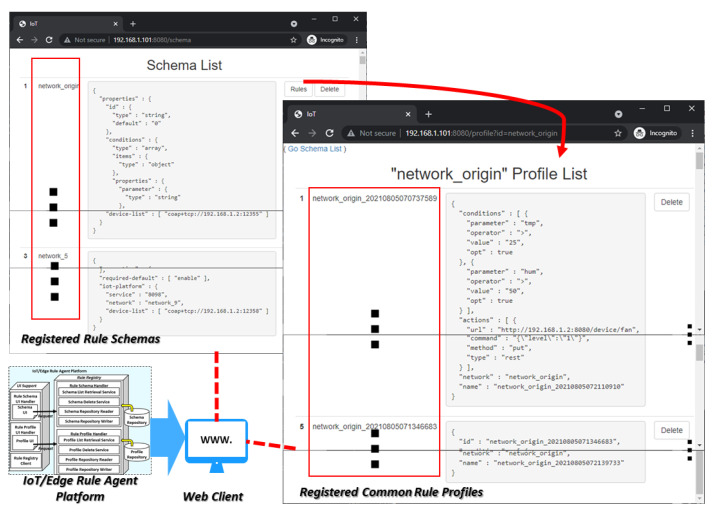
Implementation result of client service.

Figure 18 shows the implementation result of the client service that is provided by the UI support of the IERAP. For presenting the registered rule schemas and profiles, the UI support interacts with the rule registry internally based on microservices. The UI support includes the UI resources that are used for integrating the information of the retrieved rule schemas and profiles and delivered to the web client such as web browsers. Firstly, the schema list is included in the UI and presented to the users. In the list, clicking the item retrieves the rule profile list of the corresponding IoT edge network. Each item includes a function to delete it from the list. Also, by including more functions, the IERAP can provide a rich user experience to manage the rule schemas and profiles.

## 6. Performance Evaluation

For providing the performance results of the proposed IERAP, we provide initial memory usage of the deployed microservice modules, network latency and memory usage in the deploying process which can be referred to in future implementations of heterogeneous rule-enabled IoT edge networks.

**Figure 19:**
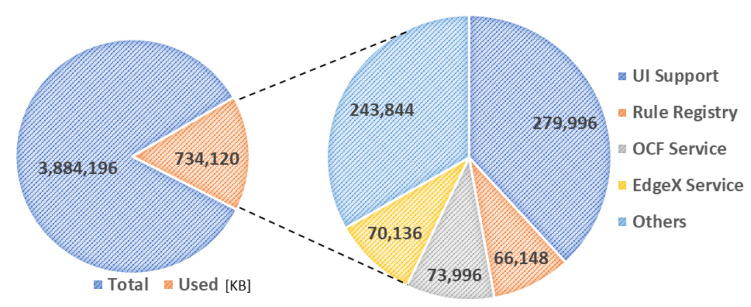
IERAP memory usage for running single device proxy.

**Figure 20:**
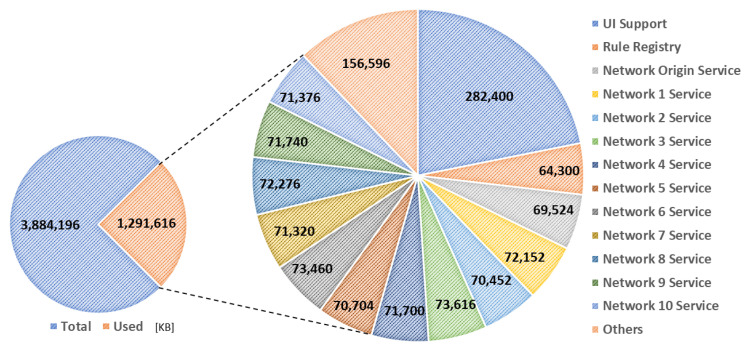
IERAP memory usage for running multiple device proxy.

Figure 19 and Figure 20 present the memory usage of the IERAP for the experiment based on a single device proxy and multiple device proxies. For the single device proxy, the OCF service is performed to forward the rule profile to the network where the OCF-based IoT edge platforms are deployed. The Raspberry Pi device provides total memory of 3,884,196 KB. The device operates the IERAP that shares 490,276 KB for the single device proxy and 1,135,020 KB for the multiple device proxies.

In the single device proxy experiment, the UI support takes 279,996 KB, which is the module sharing the most, and other modules of the IERAP share 70,136 KB, 73,996 KB and 66,148 KB, respectively.

In the multiple device proxies experiment, the UI support takes 282,400 KB, which is the module sharing the most. The rule registry shares 64,300 KB and EdgeX-based device proxy shares 69,524 KB, which is named the Network Origin Service. The OCF-based device proxies share 71,665 KB on average and 788,320 KB in total.

**Figure 21:**
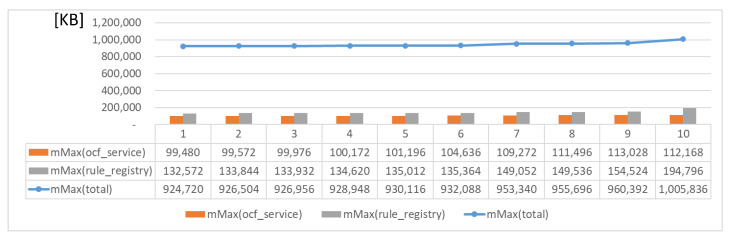
Memory usage for deploying rule profile by single device proxy.

Figure 21 shows the memory usage for deploying the rule profile by the single device proxy to multiple IoT edge platforms of a network. In this experiment, a device proxy is deployed to handle the deployment for one to ten platforms. As the number of platforms increases, the max memory usages are increased for the device proxy and rule registry. However, the changes are not obvious. The total memory usage is affected by these modules mainly. Therefore, the changes are also dependent on the memory usage changes of the device proxy and rule registry.

**Figure 22:**
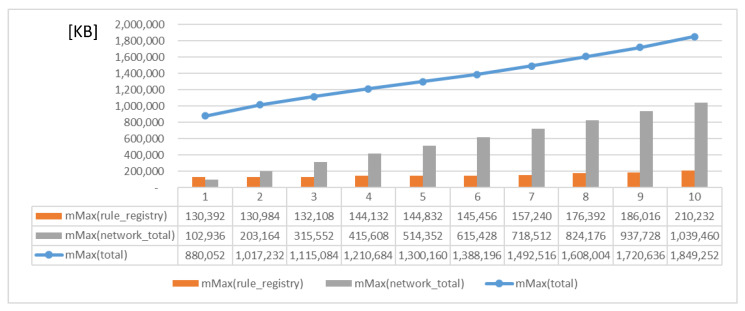
Memory usage for deploying rule profile by multiple device proxies.

Figure 22 shows the memory usage for deploying the rule profile by multiple device proxies to multiple IoT edge platforms that are deployed to multiple networks respectively. As the number of platforms increases, more device proxies are deployed for handling the corresponding deployment. Therefore, the total memory usage is obviously increased.

For providing the delays of the deployment to the platforms, the round-trip times (RTTs) are collected by calculating the difference between the request start time and response return time.

**Figure 23:**
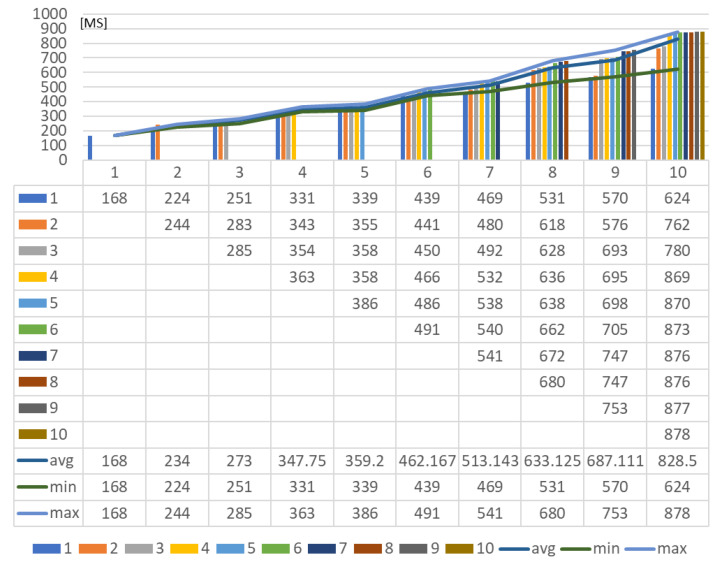
Network latency for deploying rule profile through single device proxy.

Figure 23 shows the RTTs of deploying to multiple platforms using a device proxy. With the increased number of platforms, the latency of each deployment is increased according to the averages. The requests are performed asynchronously. Therefore, the maximum latency is the total delay time for deploying to all platforms through the device proxy.

**Figure 24:**
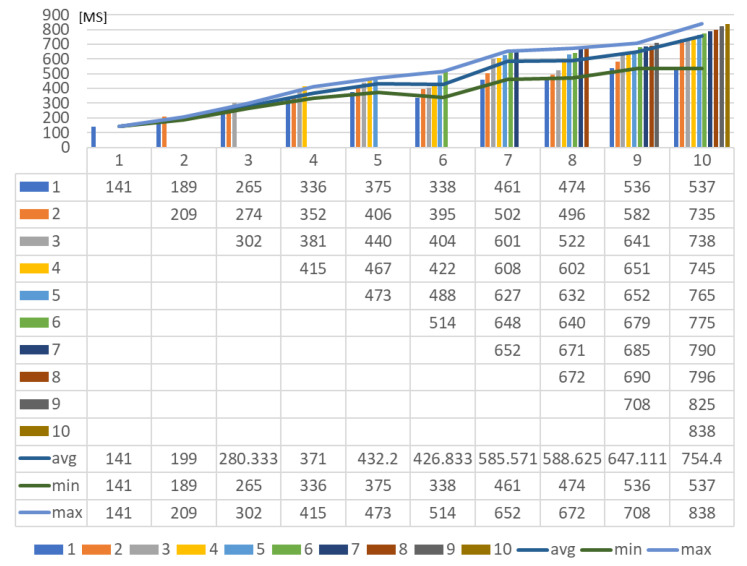
Network latency for deploying rule profile through multiple device proxies.

As shown in Figure 24, with the increased number of deployments, the network latencies are increased although more device proxies are deployed to handle the deployment asynchronously using multi-threading. This experiment shows that the device performance affects the network latency. 

The reason for the increased network latency can be explained by the device performance being affected by total memory usage and network consumption. By comparing the deployment latency of a single device proxy and multiple device proxies, the total delay of multiple deployment is more efficient by single_deployment_delay×number_of_deployment. However, for deploying to the same IoT edge network, deploying a single device proxy can be suggested due to the lower memory usage.

## 7. Conclusions and Future Directions

For operating the consistent rule scenario in the rule-enabled IoT edge architecture, the knowledge of rules must be acknowledged by the heterogeneous IoT edge devices regardless of existing platforms or newly developed emerging solutions. In this paper, we proposed transparent rule enablement based on the commonization approach by deploying the device proxies to the proposed IoT Edge Rule Agent Platform (IERAP) to share the consistent rules with IoT edge platforms without considering the difference in protocols and data formats. Each device proxy of IERAP only considers the translation of corresponding platform-specific and common formats. Also, the rules are deployed by the corresponding device proxy which enables rules to be deployed to heterogeneous IoT edge platforms to perform the consistent rule scenario without considering the format and underlying protocols of the destination platform. We developed the proposed transparent rule-enabled IoT edge networks using the international IoT edge standard frameworks including EdgeX and OCF to provide rule services in IoT edge networks. The performance results including initial memory usage of the deployed microservice modules, network latency and memory usage in the deploying process are presented and can be referred to in future implementations of the heterogeneous rule-enabled IoT edge networks.

As the future directions, we will apply IERAP to the oneM2M entities to support interworking with OCF and EdgeX platforms. Currently, the oneM2M device proxy is being developed for the Message Queuing Telemetry Transport (MQTT) architecture to enable the rule translation in the oneM2M platforms. As a part of the project, the rule enablement approach will be updated to operate a deep learning engine for providing intelligent services in the network edges. Therefore, dynamic and real-time edge services based on intelligent functions are provided without considering underlying protocols using heterogeneous IoT edge platforms.

## Figures and Tables

**Table 1:** Development Environment.

**Service Provider**	**Framework and Library**	**Platform**
Rule Registry	Jetty 9.4.40, Http Client 4.5.13, Jackson 2.11.4	Ubuntu Server 21.04 64-bit on Raspberry Pi 4 Model B
UI Support	Spring Boot 2.5.3, Http Client 4.5.13, Jackson 2.11.4	
EdgeX-Based Network Service	IoTivity 2.2.2, Jetty 9.4.40, Http Client 4.5.13, Jackson 2.11.4	
OCF-Based Network Service	IoTivity 2.2.2, Jetty 9.4.40, Http Client 4.5.13, Jackson 2.11.4	
OCF-Based Rules Engine	EdgeX Framework Hanoi with EMQ Kuiper	
OCF-Based Rule Server	IoTivity 2.2.2	Windows 10 Pro 64-bit on High-Performance Computer
Web Client	Talend API Tester	

## Data Availability

Data sharing is not applicable.
